# Purpose and Meaning in Life in Older Age: A Correlational Meta-Analysis

**DOI:** 10.21203/rs.3.rs-8930714/v1

**Published:** 2026-03-19

**Authors:** Ian Boreham, Catherine O’Gorman, Phoebe Bailey

**Affiliations:** University of Technology Sydney; University of Technology Sydney; University of Technology Sydney

## Abstract

This meta-analysis evaluated the relation between purpose or meaning in life and age across the adult lifespan. This represents the most comprehensive assessment to date of the relations between these two variables, based on sixty years of meaning and purpose research. The study included 335articles gathered through a search in PsycINFO, PsycARTICLES, and EBSCO. Using a three-level random effects model, the overall effect size based on *k* = 401 studies and *N* = 194,296 participants was *r* = .05 [95% CIs .03, .06], *p* < .001. There were significant moderating effects of type of purpose scale, age group of participants, and Hofstede’s cultural dimension of motivation towards achievement and success. Results suggest that the relationship between purpose and age may switch from positive to negative in samples excluding the emerging adult age group and including the very old. Moderating effects of type of purpose scale support recent efforts to understand the multi-dimensional nature of meaning and purpose, and highlight the differing role that motivational, cognitive and emotional aspects of meaning and purpose may play at different stages of the life course, Specifically, scales showing a greater future-focus potentially reduce the strength of the relationship between purpose and age. Building upon these collective findings we provide recommendations for theory and practice, as well as address limitations in the existing literature to guide future investigations.

## Introduction

Purpose in life or meaning in life may be strong candidates as a protective factors against some of the challenges of older age. Having a purpose is generally defined as committing to a broad life direction that organizes one’s short- and long-term goals and in turn helps guide daily behaviors (McKnight and Kashdan, 2009). Meaning in life is a broader construct, often including purpose as a component, together with the sense that life makes sense (coherence), and an evaluative component stressing the significance of one’s life (usually expressed as significance or mattering; see George & Park 2016a, 2016b; Martela & Steger 2016, 2023). A number of studies have shown links between higher purpose or meaning and improved mental and physical health (Hill, Edmonds et al., 2019; Sutin et al., 2021a, 2021b; Wood & Joseph, 2010). An early meta-analysis revealed declines in purpose from middle-age to older-age (Pinquart, 2002). However, more recent studies provide evidence for cultivation of purpose and meaning in older age (Kalafatoğlu, 2022; Ratner et al., 2022; Schnell & Danbolt , 2023). The current study uses a cross-sectional meta-analytic approach to address the question of the relationship between sense of purpose or meaning and age across the full adult age-range.

### Purpose or Meaning and Aging Trajectories

With regards to purpose or meaning in life and aging, the balance of evidence points to purpose declining in older age (Boyle et al., 2022; Clarke et al., 2000; Hill, Turiano et al., 2015; Hill & Weston, 2019; Ryff, 1989; Springer et al., 2011). Although these relationships appear to hold for cross-sectional and longitudinal studies, all studies use the same measure of purpose that has come under challenge for being too future-focused, potentially limiting the expression of a more present-focused purpose and meaning for older adults (see next section). However, several studies have found support for meaning and purpose increasing with age (Morgan & Robinson, 2013; Reker, 2005; Steger et al., 2009; Van Ranst & Marcoen, 1997). Possible explanations for this increase are varied and include an age-related shift to more intrinsically satisfying activities (Morgan & Robinson, 2013), as well as greater uncertainty in life for emerging adults, leading to less conviction surrounding the establishment and achievement of clear goals and aims (Reker, 2005). Lifespan developmental theories also provide support for the possibility of sustaining, or even increasing, meaning and purpose into older age. For example, socioemotional selectivity theory (Carstensen, 1995, 2021) proposes that with reduced perceived time remaining in life, older adults move away from future-focused knowledge and novelty-gaining goals and purposes, towards present-focused purposes driven by enhancing present emotional experience.

Research regarding purpose trajectories from younger adulthood to middle age is also mixed with support for either stability (Ryff, 1991), decreases in purpose starting early in life (Keyes, 2011; Ryff & Keyes, 1995), or increase in purpose from young adulthood to middle age, before declining in older age (Ryff, 1989; Mann et al., 2021). Meta-analysis looking at purpose in life and age relationships found a small negative association of *r* = −.12 from middle age (<60 years) to older age (> 70 years) across 48 studies, with declines in purpose shown for both middle aged adults and older adults (Pinquart, 2002). They attributed this decline to increasing losses such as widowhood and retirement. The majority of studies in Pinquart’s meta-analysis used either the Purpose in Life Scale (Crumbaugh & Maholick, 1964) or the purpose subscale of the psychological well-being scale (Ryff, 1989). In addition, contributing study numbers were small (30 or less).

### The Last Twenty Years of Purpose Research

Early measures of purpose and meaning used both terms interchangeably (Battista & Almond, 1973; Crumbaugh & Maholick, 1964), with very few separating out purpose from the broader meaning construct (exception: Peacock & Reker, 1981). These early operational efforts did separate the presence of meaning or purpose from the search for meaning, usually expressed as the strength of motivation to find meaning or purpose (Crumbaugh, 1977; Reker & Peacock, 1981). More recently, measures have arisen that either focus exclusively on purpose (Hill, Edmonds et al., 2015; Ryff, 1989; Scheier et al., 2006) or include more multi-dimensional measures of meaning that incorporate purpose (or a related construct) as one of several components (George & Park; 2016a; Krause, 2004; Martela and Steger, 2023; Morgan and Farsides, 2007). Purpose or meaning subscales also appear in broader measures of psychological, eudaimonic or existential well-being (Butler and Kern, 2016; Paloutzian & Ellison, 1982; Ryff, 1989; Waterman et al., 2010).

Since Pinquart’s meta-analysis there has been an exponential increase in purpose and meaning research, not only branching into more areas of psychological science, but also in the progression of defining the dimensionality of both meaning and purpose. A systematic review of measures of meaning in life has found over fifty valid measures (Brandstätter et al., 2012), many of which were excluded from the above-mentioned meta-analysis. Popular brief measures of purpose and meaning have emerged such as the meaning in life questionnaire (Steger et al., 2006) and the life engagement test (Scheier et al., 2006), as well as longer multi-dimensional measures including the meaningful life measure (Morgan & Farsides, 2007), and tripartite models of meaning (George & Park, 2016a; Martela & Steger, 2023). The latter argue for meaning to be composed of purpose (motivational element), coherence or comprehension (cognitive element), and significance or mattering (evaluative or affective element). With these new scales and conceptions comes growing support for a more lifespan oriented view of purpose that may be more multi-dimensional (Hill & Weston, 2019; Pfund et al., 2023), guarding against previous single-component studies using measures potentially showing age-related biases.

Pfund et al. (2023) have challenged Ryff’s purpose scale for potentially including future-dominant items that may not capture an older adults, more present-focused view of life (Carstensen, 1995; Carstensen et al., 1999). Using the Life Attitude Profile scale, Reker et al. (1987) found that scores on the present-focused life purpose subscale (measuring zest for life, contentment and discovery of a purpose) and the present and past-focused will to meaning subscale (searching and thinking about meaning, reflecting on the need for purpose throughout one’s life) increased with age. Subscales with a greater future orientation (goal seeking and future meaning) showed a near linear decrease in scores with age. Temporal focus may not be the only differentiating factor regarding age related differences in meaning or purpose. Morgan and Robinson (2013), using the Meaningful Life Measure (Morgan & Farsides, 2007), found that the dimensions of exciting life, principled life and accomplished life all showed near linear increases with age, with purposeful life and valued life showing a U-shaped trajectory, dipping in midlife and then rising or surpassing younger levels in later life. These developments and differences drive the importance of studying the moderating effect of purpose scale type on the relationship between purpose and age.

### The Current Study

The overall aim of the current meta-analysis was to use systematic review and meta-analytic techniques to analyse the relationship between purpose and age across the last sixty years of purpose research, capturing all the major measures of purpose and meaning, and spanning multiple geographical regions. Our pre-registered hypothesis was that the relationship between purpose or meaning and age, based on the mixed evidence to date, would be negligible, potentially showing a small positive association. However, it was also expected that this relationship would be moderated by the type of purpose or meaning scale used, with temporal focus potentially being a driving force. Future-dominant scales such as Ryff’s purpose scale were expected to show a more negative association between with age, as compared to other more meaning and coherence focused (i.e., temporally neutral) scales. Given the recency of tripartite models of meaning, we did not expect enough primary studies to make meaningful moderator assessments based on the tripartite components of meaning (purpose, coherence, and significance / mattering). In line with our pre-registration, further moderator analyses were planned to examine variables that may explain any within-study or between-study heterogeneity. This included sample type (healthy v clinical), region and language of sample, percentage female, percentage caucasian, age group, and Hofstede’s five cultural dimensions (Hofstede, 2011; Hofstede & Bond, 1984; The Culture Factor Group, n.d.), quality of purpose scale (core v non-core), and year of study. No predictions were pre-registered in relation to these potential moderators.

## Method

### Transparency and Openness

We adhered to the MARS guidelines for meta-analytic reporting (Appelbaum et al., 2018). All meta-analytic data, analysis code, and research materials (including our coding scheme) are available on the Open Science Framework [link removed]. Data were analyzed using R, version 4.3.1 (R Core Team, 2021) and the package *metafor*, version 3.0–2 (Viechtbauer, 2010). This review project was preregistered (link removed).

### Meta-Analysis

Our meta-analysis served to estimate the cross-sectional self-reported association between purpose or meaning in life and age. As such, every included study reported a zero-order correlation between these two variables.

### Literature Search

**Figure 1:**
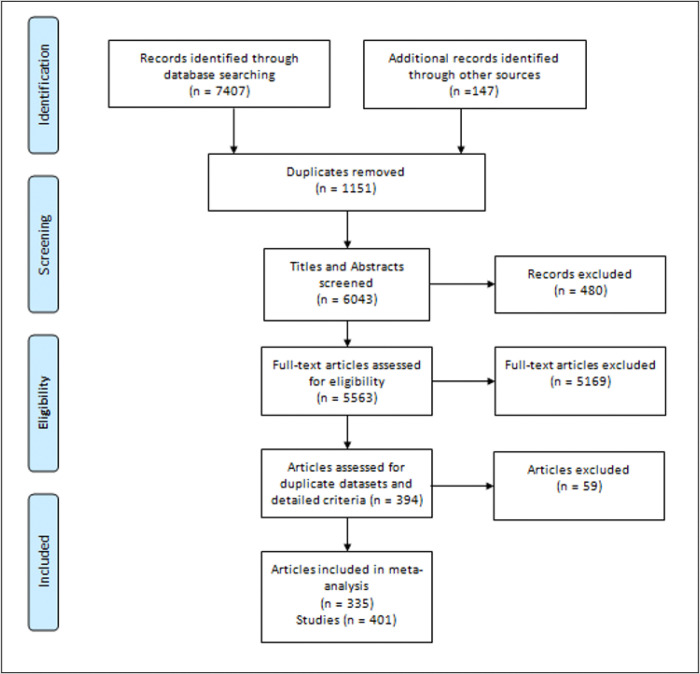
PRISMA Flow Diagram

The literature search sought to identify any study that reported a correlation between purpose or meaning in life and age as measured continuously for adult populations. The literature searches reported in this paper were conducted between July 2023 and June 2024 (see Table S1). Keyword searches were conducted in EBSCO and PsycINFO / PsychARTICLES, which included dissertations and English language articles. The search strategy was conducted in two phases with the initial phase (PsycINFO / PsychARTICLES) deliberately using broad search terms such as ‘purpose in life’ and ‘meaning in life’ to capture a broad array of potentially relevant studies (see table S1 for all exact search terms). This phase also included search terms targeted towards the more heavily used purpose and meaning scales such as the ‘meaning in life questionnaire’ and the ‘purpose in life scale’. The second phase of the search (EBSCO) used more targeted search terms aimed at identifying specific purpose and meaning scales together with the words ‘age’ and ‘correlation’. Additional references from key meta-analyses on purpose in life (Boreham & Schutte, 2023; Pinquart, 2002) were also included, as were any references from reviewed papers that appeared promising for including purpose and age related relationships. After merging the above sources and removing obvious duplicates, the combined dataset consisted of 6043 articles. Based on title screening, titles that related to adolescent studies or qualitative studies were not reviewed. Similarly, records relating to books or foreign language articles were not reviewed. In addition, several records could not be retrieved. Based on these exclusions, the full text was examined for 5563 records. A PRISMA flowchart outlines the process for selecting studies for inclusion in this meta-analysis (see Fig. 1).

### Eligibility Criteria

Several criteria needed to be satisfied for correlations to be retained in the meta-analysis. For consistency, the study needed to involve self-report measurement of either purpose or meaning in life and age. Second, the age of participants needed to include sufficient variation (minimum range of 5 years) so that the age by purpose relationship was meaningful. This age by purpose relationship had to be expressed as a zero-order Pearson correlation. In certain cases where correlational data was missing, the raw data were requested from the authors. Third, participants needed to be above 18 years of age, although in 24 studies the minimum age was below 18. These studies were included as most participants were over 18. Lastly, the measure of purpose or meaning was required to measure the presence of meaning or purpose as measured by a continuous scale. This led to the exclusion of studies that were solely measuring the ‘search for meaning’, as well as studies that modified global measures of meaning to focus on more localised interpretations. Several studies failed this last test, modifying the wording of common meaning items to assess whether the presence of meaning arose from certain specific scenarios (such as care giving or suffering from cancer). Several studies employed the popular meaning in life questionnaire (Steger et al., 2006), with most providing results for both the presence of meaning subscale and the search for meaning subscale. In these cases, correlations were only extracted for the presence of meaning subscale. However, there were a small number of studies that met all other inclusion criteria but only included results for the ‘full scale’, which combined both presence and search for meaning items. These studies have been included (see Table 2).

Based on the above criteria and initial inspection, 394 articles met the criteria. However, more detailed inspection of this study pool, in preparation for data extraction, led to the further exclusion of 59 articles. Twenty articles were excluded as they reported correlations that used a sample that overlapped with another article. In these cases, we sought to retain the article that provided the most comprehensive study in terms of sample and measurement. Six articles used data from the longitudinal U.S Health and Retirement Study (see Table S2). The HRS (Health and Retirement Study) is sponsored by the National Institute on Aging (grant number NIA U01AG009740) and is conducted by the University of Michigan. Including all six studies would have led to sample overlap and potentially biasing results, given the large sample sizes. As data was publicly available for all bi-annual HRS waves from 2006–2018, a decision was made to use the available data for greater completeness. Therefore, all six articles were replaced with a single combined ‘HRS’ study. Twelve articles used data from the National Survey of Midlife Development in the US (see Table S3). Given the common thread of participants throughout MIDUS waves I, II and III, the Homan and Kong (2020) study was retained as MIDUS II employed the 7-item version of the Ryff scale (as opposed to the less reliable 3-item version in MIDUS I) and closely resembled verifiable results from publicly available data (see OSF registry). Therefore seven studies using MIDUS data were excluded due to sample overlap. Seventeen additional studies were excluded due to concerns with how purpose or age was measured or to discrepancies between the reported age-purpose correlation and the supporting text. Eleven studies were excluded due to issues with the sample construction, with the sample either being younger than initially thought (adolescents), too small, or due to difficulty determining which subsample group the correlation belonged to. One further study was excluded due to purpose being rated by an informant (rather than self-rated). References for all excluded studies can be found in the OSF registry.

The final set consisted of 335 articles and 401 studies. Some articles reported purpose and age correlations separately for different groups (e.g., males and females), and for separate measures of purpose, and these were treated as separate studies. If a purpose scale included purpose and age correlations for both the full scale and for subscales, the full-scale correlation was extracted, with the exception being the previously mentioned meaning in life questionnaire.

### Data Extraction and Data Coding Procedures

For each included study, we extracted the following study features: year of study, sample size, purpose measure, number of items in purpose measure, internal reliability (Cronbach alpha) of purpose measure for the study, percentage female, percentage Caucasian, age range of participants, whether the age range was reported, mean age, country of sample, language of study, type of sample (e.g., clinical or healthy), the source of the correlations (e.g., from the article, provided following correspondence with author, etc.), reference details, additional notes, and the zero-order correlation between purpose and age.

In regards to data extraction and coding, the year of study was extracted as the year of publication if not explicitly stated in the text. If the data gathering exercise for a study spanned multiple years, the most recent year was chosen. Not all studies provided an age range, yet each study was coded into age range categories including emerging (E) for 18–39 years, middle (M) for 40–64 years, old (O) for 65–79 years, and very old (V) for 80 years and above. As an example, a study with an age range of 18–70 would be coded as E-O. When a study failed to provide the age range, the age range was calculated using the mean age, standard deviation and study text. For a number of these studies the study text often included clues as to the age range (e.g., middle aged or students etc.). Those studies reporting age ranges were coded as R (reported), with those not reporting age range coded as C (calculated). Studies were coded as ‘core’ if the purpose scale was a known measure and included at least 5 items. Five was chosen as this is the number of items in the well-established meaning in life questionnaire presence subscale and several studies involving purpose measures with fewer items have shown low internal consistency. In coding each study sample as clinical (C) or healthy (H), the clinical designation was reserved for those samples that had a clear physical (e.g., cancer) or mental health (e.g., major depressive disorder) diagnosis. In the cases of elderly samples, if no specific sample-wide condition was mentioned then a status of H was assigned. However, there were a small number of cases where the study text clearly outlined a dependent living status, in which case C was chosen.

All data was extracted into Excel by the first author, with each study reviewed multiple times to check for data extraction accuracy.

### Missing Data

Despite the extensive search and number of studies included in the meta-analysis, it is very difficult to identify, retrieve, and include every relevant study. In addition, there is the very real possibility that non-significant results are less likely to be published (Rosenthal, 1979). Following the process outlined by Habeck and Schultz (2015) we tested for publication bias using Egger’s regression test (Egger et al. 1997; Sterne and Egger 2005) by modifying the regression model to include the standard error of the effect sizes as a moderator. If the intercept significantly deviates from zero, the overall relationship between the precision and size of studies included in the data set is considered asymmetrical, and therefore, biased (Sterne and Egger 2005). Diagnostic tests for identifying, and rules for excluding, outliers and influential data points are still evolving for multivariate/multi-level meta-analytical models (Viechtbauer and Cheung 2010). With limited options currently available using the metafor package with the rma.mv() function, we define influential outliers as effect sizes with hat values (i.e. diagonal elements of the hat matrix) greater than two times the average hat value (i.e. influential) and standardized residual values exceeding 3.0 (i.e. outliers; Stevens, 1984; Viechtbauer and Cheung, 2010; Aguinis et al., 2013).

### Data Analytic Approach

Meta-analytic correlations were estimated using a random effects model (Raudenbush, 2009) with the metafor package in R (Viechtbauer, 2010). The random effects model assumes that the primary studies in a meta-analysis are a random sample of the population of studies. Most traditional random effects models focus on sampling variation and variation between studies (Houben et al., 2015). However, a key assumption is that included subject samples are independent (Assink et al., 2015), which is violated when multiple effect sizes are extracted from the same study sample as is the case with the current meta-analysis. Therefore, we performed a three-level meta-analysis that allows the effect sizes to be correlated within a study (Assink & Wibbelink, 2016; Cheung, 2014). This approach models three sources of variance: sampling variance of the observed effect sizes (Level 1), variance between effect sizes from the same study (Level 2), and variance between studies (Level 3) (Assink et al., 2015). The R syntax followed the guidance provided by Assink & Wibbelink (2016), using the function “rma.mv” of the metafor package (Viechtbauer, 2010) with R version 4.3.1 (R Core Team, 2021). As explained in Assink et al. (2015), all model parameters were estimated using the restricted maximum likelihood estimation method. Continuous moderator variables were mean-centred and dummy variables were created for all categorical moderators (see data files: Pur_Age_R_Data_File_v3.csv). Meta-regression models used the t distribution to test individual regression coefficients and calculate confidence intervals. The F-distribution was used to test moderator models, testing the null hypothesis that all group means are equal (categorical moderators) and the assumption of no relationship between a continuous moderator and effect sizes. Log-likelihood-ratio-tests were performed to test level 2 and level 3 variance for each model (see Assink et al., 2015; Assink & Wibbelink, 2016). The log-likelihood-ratio-tests were performed one-tailed and all other tests were performed two-tailed. We considered p-values of .05 as statistically significant. All correlations were Fisher’s Z transformed prior to running the models. For Fisher’s Z, it has been shown that the sampling variance is equal to 1/(*N* − 3), with *N* equal to the sample size (Lipsey & Wilson, 2001). This was used to derive the sampling variance for each study.

### Measures

#### Purpose and Meaning Scales

Across all studies, 29 recognised meaning and purpose scales were employed (see Table 1), with scale items reviewed for 27 of those scales (see Table 1, note 4 for the 2 scales where items were unavailable). Of the studies that employed the meaning in life questionnaire, a small number of these used the full 10-item version which sums the scores on the presence of meaning and search for meaning subscale. For the remainder, the presence of meaning subscale was used due to its focus on the existence of meaning in one’s life. If scales were measuring broader constructs (such as spirituality) then only the subscale data relating to meaning and purpose was extracted. For some scales, a deliberate decision was made to omit data relating to certain subscales that may have a claim to relating to purpose and meaning. This was the case for the studies employing the life attitude profile scale, where data was only extracted for the purpose, coherence and personal meaning index subscales (the latter being a composite score of the two previously mentioned subscales). Three of the 29 scales may be regarded as somewhat different to the rest, with both the Sources of Meaning Profile (Revised) and the Personal Meaning Profile asking participants about their engagement with ‘sources of meaning’ in their lives (such as relationships or achievement related activities). Both scales have been shown to be valid measures of meaning. The Engagement in Meaningful Activities Survey (Revised) is slightly different again, with all questions asking participants about the quality of and benefits derived from their activities (e.g., ‘the activities I do help other people’). Studies using fewer than five items to measure meaning or purpose were marked as non-core. Frequently this was where studies used a shortened version of a longer scale or where data were only available for a shorter subscale measure. Only one study failed to report the number of items in its purpose scale (Türkarslan et al., 2020). This study employed the well-established purpose in life scale which is usually employed with the full 20-item version. For this reason the study was marked as core.

#### Adhoc Purpose Measures

A small number of studies employed measures of purpose and meaning that could not be traced to established scales. For all but one of these studies, the items used were listed in the accompanying article and were reviewed for appropriateness. Some of the studies used a single item measure, usually asking directly about one’s perception of their level of purpose or meaning in life. The only studies employing ad hoc measures that were marked as core were Lee et al. (2017) and Trzebiński et al. (2020). Lee et al. (2017) employed a 5-item version reported as a modified version of the purpose in life scale. Trzebiński et al. (2020) employed an 8-item measure of purpose reported as a combination of the presence of meaning subscale from the meaning in life questionnaire and the Orientation to Life Questionnaire (Antonovsky, 1987), sometimes referred to as the Sense of Coherence scale. On verification the eight listed items did not appear on either scale, but were appropriately worded for meaning and purpose.

## Results

### Summary of the Literature

A summary of the studies included in the meta-analysis is provided in Table 2, with further details provided in the OSF repository. In total, the meta-analysis included 401 purpose and age correlations (351 core and 50 noncore) across a total sample of 194,296 participants. Non-clinical participants participated in 328 of the studies with the remaining 73 studies reporting participants with a clinical diagnosis (50 with a physical diagnosis, 17 with a mental health or substance use disorder diagnosis, and the remainder requiring general care or reporting a combined physical/mental diagnosis). The 29 recognised purpose and meaning scales spanned 388 of the 401 studies, with 13 studies employing ad hoc measures. Sixty eight percent of studies used either the meaning in life questionnaire (see Steger et al., 2006), the purpose in life scale (see Crumbaugh and Maholick, 1964), the purpose subscale from Ryff’s psychological well-being scale (see Ryff, 1989), or the Life Engagement Test (Scheier et al., 2006). Fifty-six of the studies were from prior to the year 2000, with 275 occurring after 2010, reflecting the growth in interest in purpose and meaning research. Just over 100 studies came from dissertations reflecting the breadth and depth of the literature search. Regarding age range, 147 studies included the very old (80 + years), with 102 studies only including up to old age (< 80 years), and 152 studies spanning emerging adulthood and middle age.

**Figure 2:**
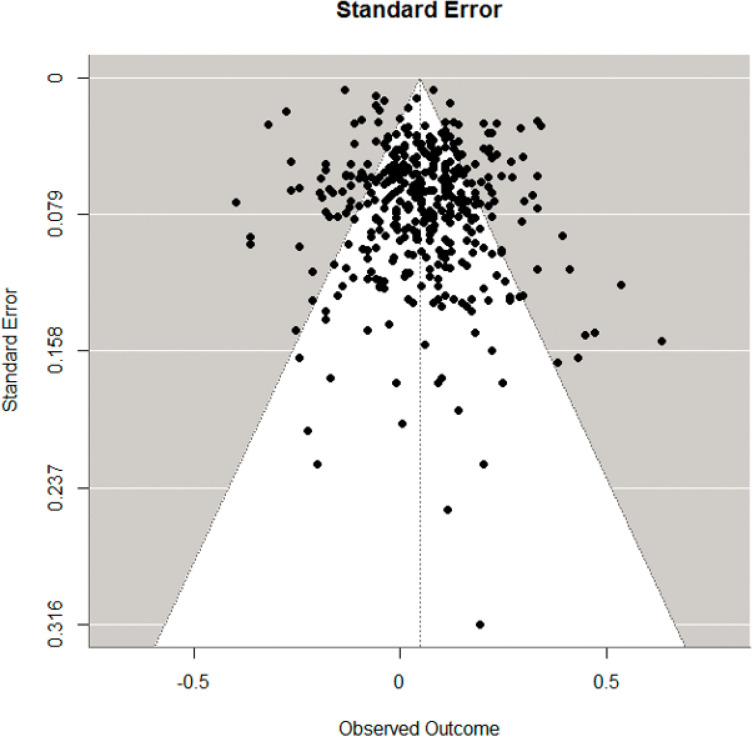
Funnel Plot

Despite relative symmetry in the funnel plot (Fig. 2), we detected publication bias in the overall data set (*p* = .034). We did not detect influential outliers in the overall data set.

### Meta-Analytic Correlations

The overall meta-analytic association between purpose in life and age was estimated by fitting a three-level meta-analytic model to the data consisting of an intercept only that represents the overall effect. The overall effect is .05 (expressed in Fisher’s *Z*_*r*_) with a standard error of .01. This overall effect is significant (*t*(400) = 6.50, p < .001). Expressed as a zero-order correlation, the overall effect is *r* = .045 [95% CIs .03, .06], *p* < .001, indicating that across samples higher purpose in life was significantly associated with increasing age, with the overall effect size in the small range. A forest plot showing effect sizes and confidence intervals for all 401 studies is shown in the supplementary materials (see Figure S1). The results for the test for heterogeneity reveal significant variation between all effect sizes, *Q*(400) = 3092.15, *p* < .001. The significance of the between study variance was tested by comparing the fit of the original three-level model with the fit of a two-level model with between study variance fixed at zero. Table 3 shows that between study variance is significant (*p* < .001). Following formulas outlined by Cheung (2014) and R syntax provided by Assink and Wibbelink (2016), the distribution of variance across the three levels of the meta-model was tested. Results showed that 13.46 percent of the total variance can be attributed to variance at level 1 (i.e., the typical within-study sampling variance); 0.28 percent of the total variance can be attributed to differences between effect sizes within studies at level 2 (i.e., within study variance); and 86.25 percent of the total variance can be attributed to differences between studies at level 3 (i.e., between-study variance). Moderator analysis was performed in order to examine variables that may explain the significant between-study variance.

### Moderator Analyses

#### Categorical Moderators with two Categories

Omnibus tests based on the F distribution (using the Knapp and Hartung adjustment; Knapp & Hartung, 2003) were employed separately for each moderator. There were no significant moderators of the overall effect by health status (healthy: *k* = 328; clinical: *k* = 73), *F*(1, 399) = 0.69, *p* = .407, core measure (core: *k* = 351; non-core: *k* = 50, *F*(1, 399) = 0.90, *p* = 0.345, language (English: *k* = 266; other: *k* = 135), *F*(1, 399) = 0.44, *p* = 0.506, or data source (journal: *k* = 296; other: *k* = 105), *F*(1, 399) = 3.58, *p* = 0.059. Mean effects and confidence intervals for all of these categories are provided in Table S4.

#### Categorical Moderators with Multiple Categories

Type of purpose scale moderator categories were based on core (five or more items) purpose scales that were used in five or more studies. All other studies were grouped under the ‘other’ category. The results of the omnibus test demonstrate that type of purpose scale moderates the overall association between age and purpose, *F*(9, 391) = 3.28, *p* < .001, with regression coefficients (Table 4) for both the Meaning in Life Questionnaire (presence subscale) and the Sources of Meaning (meaningfulness subscale) deviating significantly from zero, with mean effects for both significantly higher than the mean effect of the ‘other’ category (mean effects calculated by adding the regression coefficient to the intercept, MLQ-P: *k* = 92, *Z*_*r*_ = .08; SoMe-M: *k* = 5, *Z*_*r =*_ .19). Mean effects for all scale categories are shown in Table 5.

As per the pre-registration, age group moderator categories were analysed if they included five or more studies. The emerging to very old category was used as the reference group for regression analysis as this category spanned all ages. The results of the omnibus showed a moderating effect for age group, *F*(7, 393) = 10.36, *p* < .001. Regression models (Table 6) showed that emerging to very old (E-V, *k* = 72), middle to very old (M-V, *k* = 53), and old to very old (O-V, *k* = 23) were all significant predictors of the association between age and purpose (E-V: *t*(393) = 4.46, *p* < .001; M-V: *t*(393) = −4.56, *p* < .001; O-V: *t*(393) = −5.0, *p* < .001). Mean effects of all age groups (Table 7) ranged from − .09 (old to very old) to .10 (emerging to old, *k* = 89). Mean effects for five of the eight age groups differed significantly from zero (*p* < .05). Perhaps of greatest interest is the change in direction of the effect from positive to negative in groups that included the very old and excluded the emerging. The effect for the middle to very old (M-V, *k* = 53) and old to very old (O-V, *k* = 23) groups was negative and significant (M-V: *Z*_*r*_ = − .04 [95% CIs − .08, − .01], *p* = .021; O-V: *Z*_*r =*_ −.09 [95% CIs − .15, − .04], *p* = .001).

#### Continuous Moderators

Neither year of study, *F*(1, 399) = 2.07, *p* = .151, nor percentage female, *F*(1, 386) = .03, *p* = .85, were significant moderators. Similarly, power distance, *F*(1, 390) = .17, *p* = .676, individualism, *F*(1, 390) = .97, *p* = .325, uncertainty avoidance, *F*(1, 390) = .03, *p* = .869, and long term orientation, *F*(1, 390) = .50, *p* = .481, were not significant moderators. However, motivation towards achievement and success was a significant moderator, *F*(1, 390) = 8.37, *p* = .004. The regression coefficient is positive (*B* = .00, *t*(390) = 2.89, *p* = .004), indicating that the higher a country scores on motivation towards achievement and success, the higher the reported effects in the primary studies. Similarly, indulgence was a significant moderator, *F*(1, 390) = 5.83, *p* = .016. The regression coefficient is also positive (*B* = .00; *t*(390) = 2.42, *p* = .016), indicating that the higher a country scores on indulgence, the higher the reported effects in the primary studies.

#### Multiple Moderator Model

The multiple moderator model examines the unique effects of variables that have been identified as significant moderators in univariate analysis (Assink & Wibbelink, 2016), by extending the meta-analytic model by adding such variables. Therefore the meta-analytic model was extended with the variables; purpose scale type, age group, geographical region, Hofstede’s motivation towards achievement and success, and Hofstede’s indulgence.

The overall model is significant (*F*(22, 369) = 5.87, *p* = < .001), with significant unexplained variance remaining, *QE*(369) = 1449.84, *p* < .001. The Meaning in Life Questionnaire (presence subscale) and the Sources of Meaning (meaningfulness subscale) remained significant predictors (MLQ-P: .*B* = 041, *t*(369) = 2.63, *p* = .009; SoMe-M: *B* = .12, *t*(369) = 2.12, *p* = .035), which implies that both moderators are robust in that they are not confounded by other variables in the model. Of particular interest, the Ryff scale in the multiple moderator model also became a significant predictor (*B* = − .04, *t*(369) = −2.44, *p* = .015). The fact that the regression coefficients for both the MLQ-P and Ryff are equal and opposite is of particular note, with the former showing that reported effects in the primary studies are higher when the MLQ-P is used (relative to ‘other’ scales), and lower when the Ryff scale is used (relative to ‘other’ scales). Relative to the emerging to very old age group, the same four age groups remained significant predictors in the multiple moderator model (Table 10), with higher reported effects in primary studies for the emerging to middle and emerging to old groups, and lower reported effects for the middle to very old and old to very old groups. No geographical region category held a unique moderating effect in the multiple moderator model. Of the two Hofstede cultural dimension categories, only motivation towards achievement and success remained significant in the multiple model.

## Discussion

The present study aimed to assess the relationship between purpose or meaning in life and age across the adult lifespan. The results supported the pre-registered hypothesis, revealing a small positive relationship across 401 studies spanning sixty years of purpose and meaning research and all continents. This represents a significant update to Pinquart’s previous meta-analysis which suggested a small effect of declines in purpose with older age. Also in line with the pre-registered hypotheses was the fact that the type of purpose or meaning scale showed a significant moderating effect. Exploratory analysis revealed that the age group of the participants, and Hofstede’s cultural dimension of motivation towards achievement and success were significant moderators of the relationship between age and purpose or meaning. There were no significant differences in the effect sizes of primary studies for clinical versus healthy participants, core versus non-core measures of meaning and purpose, English versus non-English language, and journal articles versus other sources. In addition, the year of study and the percentage female within each sample showed no moderating effects.

The finding that type of scale employed had an (albeit small) effect on the relationship between purpose or meaning and age is an important finding. It supports the notion that not all purpose and meaning scales are created equal, with potentially factorial, temporal and sub-dimension differences. These differences have been commented on substantially within the literature (Hicks & King, 2009; George and Park, 2016a; Leontiev, 2013, Martela & Steger, 2016). The present study found that the 5-item presence of meaning scale (MLQ-P) and the 5-item sources of meaning – meaningfulness scale (SoMe-M) both led to an elevated and positive purpose/meaning and age correlation when compared to primary studies using scales deemed as other. In contrast, the Ryff purpose scale had the effect of reducing the overall correlation between purpose/meaning and age, with a mean effect of essentially zero across all studies, supporting the pre-registered hypothesis that scales with a greater future-focus would potentially reduce the purpose-age relationship. This is further support for socioemotional selectivity theory (Carstensen, 1995, 2021, Carstensen et al., 1999) and the previously mentioned arguments by Pfund et al. (2023) suggesting that certain items favouring a future-focus (e.g., “I live life one day at a time and don’t really think about the future”, and “I tend to focus on the present, because the future nearly always brings me problems.”), may be failing to capture a more present-focused perspective of meaning and purpose as experienced by older adults. However, these findings need to be balanced in light of the discovery that the age to meaning/purpose relationship is stronger (more positive) for countries scoring higher on motivation towards achievement and success. It may be that societies driven by achievement and success, such as the USA, facilitate a culture of continued striving for achievements that may even outlast organisational life and support the continuation of more personal purpose and meaning endeavours into older age.

In the new language of the tripartite model of meaning (George and Park, 2016a; Martela & Steger, 2016), the effect of scale type might also be attributed to dimensions of coherence/comprehension and significance/mattering. It is clear that both the MLQ-P and the SoMe-M possess a higher proportion of items relating to comprehension and coherence (e.g., MLQ-P: “I understand my life’s meaning.”; SoMe-M: “I think my life has a deeper meaning.”). In addition, although both the MLQ-P and Ryff’s scale focus on purpose and purposeful living, they do so in different ways, with the former leaving the participant to define purpose (“my life has a clear sense of purpose.”) and the latter choosing to align purpose with goals and accomplishments (e.g., “I know what I want to accomplish in my life.”, and “I used to set goals for myself, but that now seems like a waste of time.”). Regarding significance/mattering the SoMe-M items (e.g., “I feel part of a bigger whole”, and “I lead a fulfilled life”) show strong alignment to these concepts. Looked at holistically and the differences start to magnify, with scales focused on broader, and more temporally-integrated conceptions, such as a sense of understanding of one’s life, the perception that one’s life matters in the grander scheme of things, and a definition of purpose free of interpretation.

Leontiev (2017) comments on the alignment of the tripartite model of meaning with previously identified cognitive, motivational and emotional components of meaning (Reker & Wong, 1988). In this model comprehension and coherence represent cognitive activity, with mattering and significance being an emotional concern, and purpose acting as a motivational vehicle. The view that one’s emotional life becomes an increasingly important element of psychological well-being into advancing age is already well supported in the literature, most prominently by socioemotional selectivity theory (Carstensen, 1995, 2021, Carstensen et al., 1999). This aligns well with the notion that mattering and significance play an expanding role throughout aging in preserving a sense of meaning and purpose. Less established is the finding that comprehension and coherence may also play a critical role in preserving meaning and well-being into advanced age.

Coherence plays a key role in Antonovsky’s work on health promotion by drawing on order and understanding as a coping mechanism for potential stressors (Antonovsky, 1987). The Meaning Frame Theory (Noguchi, 2020) highlights the need for a frame of reference in order for someone or something to be considered meaningful. These frames may refer to the self, others (in a broad sense meaning outside of the self), and/or a purpose. Meaning in the self-frame is developed by coherence through self-integrity or identity. This may provide an important reference for older adults who may be better equipped to derive meaning through reflections on narrative self-integrity and authentic living. The ‘outside-the-self’, emotional frame may provide an alternative way to look at some of the findings from Socioemotional Selectivity Theory, suggesting that significance and mattering may be one such vehicle for achieving psychological well-being. Meaning as sense making is the core of the Meaning Maintenance Model (Proulx & Inzlicht, 2012) which links meaning to the degree to which relationships between events, objects, and experiences are as expected.

If cognitive mechanisms are propping up purpose and meaning into older age, then there may be fragility to such foundations as cognitive abilities inevitably decline at some point. Indeed, in age group moderation, multivariate analysis showed that effect sizes were significantly reduced (and negative) for both the middle to very old and old to very old categories, compared with the emerging to very old categories. Further, the age and purpose/meaning association changed from positive to negative for these two age groups that included the very old and excluded the emerging age bracket. The question of which cognitive abilities underpin a sense of comprehension and coherence is an interesting one. If sense making is reliant on mental representations of expected relationships (Proulx & Inzlicht, 2012), and compensatory efforts to restore such, when violated, then future research should examine the role of relational thinking and reasoning (Alexander, 2016) to understand the neural and cognitive underpinnings of meaning as comprehension. Relational thinking involves perceiving patterns (Alexander, 2016), with relational reasoning harnessing this pattern recognition for higher cognitive purposes. One of these purposes may include generating a sense of coherence in life, by reflecting on relationships that include temporal reflections (past to present consistency in terms of self-integrity), current interpersonal relationships, as well as fitting current circumstances into well-understood schemas. Relational reasoning studies involving visuo-spatial and analogous reasoning tasks have shown that increasingly abstract processing leads to a posterior-to-anterior recruitment shift in the pre-frontal cortex (Krawczyk et al., 2011). Evidence of age-related deficits in relational processing via analogical reasoning is mixed (Byczewska-Konieczny et al., 2019), with declines usually attributed to broader well-understood cognitive declines in processing speed, inhibitory control and working memory (Byczewska-Konieczny et al., 2019; also see Zanto & Gazzaley, 2019 for aging of the frontal lobe). Further work is needed to understand the cognitive and neural foundations of meaning as comprehension, and how this relates to cognitive aging.

### Strengths, Limitations, and Future Directions

There are some significant strengths of the current study. It is the first to synthesize the meaning and purpose literature, across the full sixty year history, as it relates to aging, with a consideration for both methodological and theoretical moderators. Also the large number of primary studies can offer confidence in the current findings. However, several limitations should also be noted. Overall, despite the broad geographical coverage, over 50% of studies included in the analysis were from U.S. samples which may limit the generalizability of the findings to other regions. Additionally, the variables assessed as moderators explained only a portion of between-study variance. Given the cross-sectional and linear nature of the study firm conclusions cannot be drawn about the longitudinal developmental trajectory of purpose and meaning in life or whether that trajectory is better served by non-linear models. There is some evidence from the current study that levels of meaning and purpose may start to decline towards older age, with this age related decline happening at more advanced ages than previously reported. Similarly, despite year of study not being a significant moderator, cohort effects cannot be ruled out in this study. One possible explanation for the difference in results of this study versus previous meta-analysis (Pinquart, 2002) may be due to cohort effects, whereby younger adults are reporting lower purpose or meaning scores in more recent times, potentially due to societal changes that have increased financial burdens on younger adults (housing and education price rises) and led to increases in mental health concerns among this age group.

The current findings offer a number of promising avenues for future research. Firstly, this study provides support for the multi-dimensional nature of meaning and purpose, and recent efforts to move towards such a model. Studies that address lifespan differences in various facets of meaning and purpose will be critical for developing a more nuanced understanding of the association between age and purpose. Results of the current study also highlight an opportunity to explore purpose and meaning in advanced age from a cognitive perspective, uncovering potential links between cognitive and brain changes and aspects of meaning and purpose. Lastly, the breadth of the measures of meaning and purpose used in the current study raise questions as to the finality of tripartite models, suggesting that more work is needed to derive a fuller picture of this construct.

## Conclusion

The last sixty years of purpose and meaning research have established meaning and purpose as a robust protective factor against mental ill-health and the challenges of aging. As the global population continues to age, it is imperative that health-promoting strategies expand beyond the well-established pillars of physical exercise, healthy nutrition, stress management and adequate sleep. Focusing on purpose and meaning with its strong links to these foundational health-pillars and markers of cognitive and functional ability is a promising strategy for aging communities and populations. In a broader sense, purpose and meaning research still lags behind personality research, in terms of universally agreed structures and measures, with some progress made in the last decade to present a tripartite model of meaning. The results of our study are encouraging from an aging perspective. While the Pinquart (2002) meta-analysis found declines in purpose for both middle-aged adults and older adults, the current data suggest that the purpose-age relationship does not become negative until in very old age, and even then the effect size is small. This suggests that overall, purpose and meaning can potentially be sustained into the latter stages of life. Emotional and cognitive interpretations of meaning may become more salient in older age, and may present exciting avenues for future research, as well as providing direction for future clinical and practical applications to enhance overall well-being.

## Figures and Tables

**Table 1:** Purpose and Meaning Measures

Measure Name	Measure Reference	Subscales used	Short Codes Used	Studies	Scale items (range)
Meaning in Life Questionnaire	Steger et al. (2006)	Presence of meaning	MLQ-P and MLQ-F (full scale)	117	1–10
Purpose in Life Scale^1^	Crumbaugh and Maholick (1964)		PiL	84	3–20
Psychological Wellbeing Purpose Subscale^2^	Ryff (1989)		Ryff	76	3–20
Spiritual Wellbeing Scale	Paloutzian and Ellison (1982); Ellison (1983)	Existential Wellbeing	SWBS-EWB	21	4–10
Life Attitude Profile (and Revised version)	Reker and Peacock (1981); Reker (2001)	Purpose, Coherence and Personal Meaning Index	LAP-P, LAP-C, LAP-PMI, LAPR-P, LAPR-C, LAPR-PMI	20	3–48
Life Engagement Test	Scheier et al. (2006)		LET	16	6
Life Regard Index	Battista and Almond (1973)	Framework	LRI, LRI-FR	11	5–28
Multidimensional Existential Meaning Scale	George and Park (2016b)	Comprehension, Purpose, and Mattering	MEMS, MEMS-C, MEMS-P, MEMS-M	9	5–15
Krause Meaning in Life Scale	Krause (2004)		Krause	8	4–8
Functional Assessment of Chronic Illness Therapy-Spiritual Well-Being Scale	Peterman et al. (2002)	Meaning	FACIT-Sp-M	7	4
The Sources of Meaning and Meaning in Life Questionnaire	Schnell (2009)	Meaningfulness	SoMe-M	5	5
Meaningful Life Measure	Morgan and Farsides (2007)	Principled Life, Purposeful Life, Valued Life	MLM, MLM-Pr, MLM-Pu, MLM-V	4	4–23
Three-Dimensional Meaning^3^	Martela and Steger (2016)	Coherence, Purpose, and Significance	3DM-C, 3DM-P, 3DM-S	3	4
Geriatric Suicide Ideation Scale	Heisel and Flett (2006)	Perceived Meaning in Life	GSIS-M	3	8
Meaning in Life Scale	Jim, Purnell et al. (2006)		MiLS	3	21–25
Robbins & Francis Purpose-in-Life Scale	Robbins and Francis (2000)		R&F	3	12
Satisfaction and Frustration of Purpose Scales	Mogollon (2021)	Satisfaction of the Need for Purpose	SFPS-S	3	6–16
Meaning in Life Measure	Hill et al. (2019)	Experience	MILM-E	2	4
Psychosocial Inventory of Ego Strengths	Markstrom et al. (1997)	Purpose	PIES-P	2	8
Claremont Purpose Scale	Bronk et al. (2018)		CPS	2	12
Meaning in Life Scale	Warner (1986); Warner and Williams (1987)		ML	2	15
The NIH Toolbox Emotion Battery^4^	Salsman et al. (2013); Salsman et al. (2014)	Meaning and Purpose	NIHTB-MPS	2	6–18
Brief Stress and Coping Inventory^4^	Rahe and Tolles (2002)	Purpose and Connection	BSCI-P	1	8
The Existential Loneliness Questionnaire^5^	Mayers et al. (2002)	Meaning in Life	ELQ-M	1	7
Engagement in Meaningful Activities Survey (Revised)	Goldberg et al. (2002); Eakman (2012)		EMAS-R	1	12
Expressions of Spirituality Inventory	MacDonald (1997)	Existential Wellbeing	ESI-EWB	1	5
Multidimensional MIL Scale	Costin and Vignoles (2020)	MIL Judgments	MMILS-MIL	1	4
Personal Meaning Profile	Wong (1998)		PMP	1	39
Sources of Meaning Profile (Revised)	Reker and Wong (1988); Reker (1996)		SOMP-R	1	17

Notes:

1. Scale available in Wheaton (1972).

2. Full 20-item version of scale received directly from Carol Ryff.

3. Scale retrieved from Morse (2021).

4. Scale items not reviewed.

5. Scale items retrieved from van Tilburg (2021)

**Table 2:** Summary of Studies Included in Meta-Analysis

Study	N	Measure	Items	F	Age	Health	Country
Abikoye and Lawal (2023)	406	MLQ-F	10	34.0	E-O	H	NG
Adams et al. (2014)	70	FACIT-Sp-M	4	74.3	E-O	H	US
Aglozo et al. (2021)	235	MLQ-P	5	56.2	M-V	H	GH
Ajayi and Ukpoju (2022)	96	MLQ-F	10		M-V	H	NG
Alexander (2015)	527	MLQ-P	5	67.0	F	H	US
Allan et al. (2016)	376	MLQ-P	5	55.6	E-O	H	US
Almeida et al. (2020)	103	PiL	20	78.6	E-O	C	PT
Alshraifeen et al. (2020)	202	SWBS-EWB	10	36.6	F^1^	C	JO
Appel et al. (2020)	189	MLQ-P	5	78.3	E	H	US
Ardelt (2016)	123	PiL	3	71.5	M-V	H	US
Ardelt and Edwards (2016)	197	PiL	3	70.3	M-V	H	US
Arends et al. (2013)	298	Ryff	6	62.0	F	C	NL
Au et al. (2023)	141	Ryff	7	77.3	E-M	H	AU
Aviad and Cohen-Louck (2021)	195	PiL	18	63.1	O-V	H	IL
Ávila and Sanjuán (2018)	175	Ryff	5	18.3	E-O	H	ES
Azañedo et al. (2021)	1494	Ryff	5	56.7	E-O	H	ES
Badia (2019)	352	MLQ-P	5	67.9	F	H	US
Bai et al. (2018)	102	FACIT-Sp-M	4	61.8	E-O	C	US
Bakhshandeh Bavarsad and Stephens (2024)	2869	LET	6	55.9	M-O	H	NZ
Bamonti (2015)	82	GSIS-M	8	64.6	M-V	H	US
Bamonti and Fiske (2021)	82	GSIS-M	8	64.6	M-V	H	US
Bassi et al. (2013)	85	Ryff	3	45.0	E-M	H	IT
Bauger et al.(2021)	219	MLQ-P	5	60.0	F	H	US
Baum (1988) Females	27	PiL	7	100.0	O-V	H	US
Baum (1988) Males	23	PiL	7	0.0	O-V	H	US
Baum and Stewart (1990)	185	PiL	7	60.5	F^2^	H	US
Beaumont and Scammell (2012)	270	MLQ-P	5	71.1	E^2^	H	CA
Beaumont and Scammell (2012)	270	ESI-EWB	5	71.1	E^2^	H	CA
Ben-Ari et al. (2012) Grandmothers	152	PiL	20	100.0	M-O	H	IL
Ben-Ari et al. (2012) Mothers	152	PiL	20	100.0	E	H	IL
Bergman and Bodner (2022)	318	Krause	8	47.5	O-V	H	IL
Bergman et al. (2018)	268	MLQ-P	3	73.5	E-O	H	IL
Berrios et al. (2018) Study 1	429	MLQ-P	5	68.8	E-M	H	GB
Birichi (2015)	226	Ryff	7	0.0	E-M	H	US
Bobkowski (2010)	573	Ryff	3	46.8	E	H	US
Bodner et al. (2021)	309	Krause	8	48.9	M	H	IL
Boreham et al. (in press)	312	Ryff	20	49.0	F	H	I
Boreham et al. (in press)	312	LET	6	49.0	F	H	I
Boreham et al. (in press)	312	LRI	28	49.0	F	H	I
Boyle et al. (2022)	2558	Ryff	10	75.7	M-V	H	US
Boyraz and Lightsey (2012)	232	MLQ-P	5	78.4	E-O	H	US
Braam (2006)	928	LRI-FR	5	38.9	O-V	H	NL
Bronk et al. (2018)	241	CPS	12	42.9	E	J	US
Bronk et al. (2018)	241	PiL	20	42.9	E	J	US
Bruce (1996)	101	SWBS-EWB	10	47.5	E-O	H	US
Bryan et al. (2013)	273	MLQ-P	5	18.3	E-M	H	US
Bryan et al. (2019)	997	MLQ-P	5	15.9	E-M	H	US
Burrow and Rainone (2017)	300	LET	6	49.0	E-O	H	US
Burrow and Spreng (2016)	503	NIHTB-MPS	18	59.0	E	H	US
Burrow et al. (2014)	205	Ryff	9	62.0	E-O	H	US
Cabness (2003)	19	PiL	20	79.0	M-V	H	US
Carpenter (1996)	128	Ryff	14	100.0	E-O	H	US
Castillo Mathis (2023)	147	MLQ-P	5	63.0	M-O	H	US
Chan et al. (2012)	386	LET	6	100.0	E-M	H	US
Chang et al. (2021)	648	MLQ-P	5	49.2	E	H	CN
Chasson et al. (2021)	1138	MLQ-P	5	100.0	E	H	IL
Chow (2017)	192	ML	15	38.0	M-V	C	HK
Chow and Ho (2012) Care givers	132	PiL	20	61.4	M-V	H	HK
Chow and Ho (2012) Care recipients	132	PiL	20		M-V	C	HK
Chukwuorji et al. (2019)	809	MLQ-P	5	49.3	F^3^	H	NG
Cicone (2003)	209	Ryff	9	84.0	E-O	H	US
Clement (2018)	72	MLQ-P	5	53.5	M-V	C	US
Clement (2018)	72	MEMS-C	5	53.5	M-V	C	US
Clement (2018)	72	MEMS-M	5	53.5	M-V	C	US
Clement (2018)	72	MEMS-P	5	53.5	M-V	C	US
Coleman (1996)	117	SWBS-EWB	10	16.2	E-O	C	US
Corral-Frías et al. (2019)	179	Ryff	6	80.7	E-M	H	MX
Costanza et al. (2020)	199	MLQ-P	5	60.3	F^4^	C	CH
Coward (1996)	145	PiL	20	72.0	F	H	US
Cranford et al. (2014)	364	PiL	20	44.0	E-O	C	US
Crea and Francis (2022)	156	R&F	12	39.1	F	H	IT
Creedon (2013)	187	MLQ-P	5	72.0	F	H	US
Crego et al. (2020)	1628	MLQ-P	5	87.3	E-O	H	I
Crego et al. (2021)	1267	Ryff	5	100.0	E-O	H	I
Czekierda et al. (2019)	339	Other	1	57.9	F	C	PL
Dakin et al. (2021) Study 1	196	MLQ-P	5	43.4	E-O	H	US
Dakin et al. (2021) Study 2	191	MLQ-P	5	44.5	E-O	H	US
Dakin et al. (2021) Study 3	195	MLQ-P	5	45.1	E-O	H	US
Dakin et al. (2021) Study 5	370	MLQ-P	5	40.5	E-O	H	US
Damiani et al. (2023)	278	Ryff	14	69.8	E-M	C	US
Davies et al. (2022)	174	MLQ-P	5	44.2	E-O	H	US
de Miguel et al. (2018) LGB	209	Ryff	14	41.6	E-M	H	ES
Dellaria (2017)	736	SWBS-EWB	10	33.6	F	H	US
deRoon-Cassini et al. (2009)	79	PiL	20	4.0	F	C	US
Dewitte et al. (2022) T1	140	MLQ-P	3	76.4	O-V	C	BE
Dewitte, Vandenbulcke, et al. (2021) Study 1	275	MLQ-P	3	70.8	O-V	H	BE
Dewitte, Vandenbulcke, et al. (2021) Study 2	94	MLQ-P	3	77.9	O-V	C	BE
Dodd (2002)	77	SWBS-EWB	10	5.2	E-O	H	US
Drebing (1990)	89	PiL	20	100.0	E-M	H	US
Duke (1977) Males	64	PiL	8	65.6	E-O	C	US
Eakman et al. (2010)	158	PiL	20	77.8	O-V	H	US
Erci (2015)	182	LAP-P	8	41.2	F	C	TR
Erci (2015)	182	LAP-C	7	41.2	F	C	TR
Farrell (2013)	240	FACIT-Sp-M	4	55.8	F	C	US
Feder et al. (2008)	30	Ryff	7	0.0	M-O	H	US
Feder et al. (2013)	198	Ryff	8	19.5	E-O	H	PK
Ferguson et al. (2017)	259	MLQ-P	5	55.2	O-V	H	AU
Fike et al. (2022)	171	SWBS-EWB	10	0.0	E-O	H	US
Fletcher (1981)	82	PiL	20	100.0	O-V	H	US
Flood (2007)	147	PiL	20	76.3	M-V	H	US
Fortems et al. (2022)	96	MLQ-P	5	65.6	E-M	H	BE
Fox (2001)	75	LRI-FR	14	66.7	F	C	US
Francis and Crea (2016)	155	PiL	20		E-O	H	IT
Francis and Crea (2016)	155	R&F	12		E-O	H	IT
Francis et al. (2010)	407	R&F	12	66.0	M-V	H	GB
Fraser et al. (2023)	492	MLQ-P	5	53.8	F	H	CA
Freeman (1996)	133	SWBS-EWB	10	0.0	E-M	H	US
G. Wang et al. (2023)	593	MLQ-P	5	46.4	E^4^	H	CN
Galek et al. (2015)	1453	Other	2	53.0	F	H	US
Ganocy et al. (2016)	398	SWBS-EWB	10	12.0	E-M^2^	H	US
García-Alandete et al. (2018) Females	224	PiL	20	100.0	E	H	ES
García-Alandete et al. (2018) Males	125	PiL	20	0.0	E	H	ES
García-Alandete, Ros, et al. (2018)	250	PiL	19	100.0	E-M^3^	C	ES
Giannone et al. (2019)	353	PiL	20	75.0	E	H	US
Goldenthal (1981)	49	PiL	20	0.0	E-O	C	US
Golovchanova et al. (2021)	145	MLQ-P	5	62.1	E-M^2^	C	BE
González-Cutre et al. (2020) Study 2	598	MLQ-P	5	54.2	E-O	H	ES
Goodman et al. (2019) Study 1	1789	MLQ-F	10	100.0	E-M	H	KE
Goodman et al. (2019) Study 2	263	MLQ-F	10	0.0	E	H	KE
Goodman et al.(2023)	335	MLQ-P	3	94.0	E-O	H	KE
Goodwill and Hope (2024)	375	MLQ-P	5		E	H	US
Graeser (1994)	105	PiL	20	0.0	E-M	C	US
Grimm (2005)	477	SWBS-EWB	10	75.1	F	H	US
Gutierrez (1992) Females	87	PiL	20	100.0	M-V	H	US
Gutierrez (1992) Males	13	PiL	20	0.0	M-V	H	US
H. Zhang et al. (2018)	1063	MLQ-P	5	42.0	E	H	CN
Haase et al. (2012)	374	Ryff	9	70.1	E-M	H	DE
Haase et al. (2021) T1	164	LET	6	62.1	E	H	CA
Halama et al. (2024)	1368	MLQ-P	5	51.2	F	H	SK
Hamm et al. (2022)	292	NIHTB-MPS	6	50.0	E-O	H	US
Han et al. (2022)	*3893*	Other	1	51.5	M-V	H	JP
Hanna and Strober (2020)	186	Ryff	14	90.0	E-M	C	US
Harshaw (1999)	114	PiL	20	68.4	E-M	H	US
Health and Retirement Study (2018)	21517	Ryff	7	58.2	F	H	US
Heidrich (1993)	240	Ryff	20	100.0	O-V	H	US
Heidrich et al. (1994)	108	Ryff	15	63.0	F	C	US
Helminiak (1994) Females	318	PiL	20	100.0	M	H	US
Helminiak (1994) Males	279	PiL	20	0.0	M	H	US
Henderson-King and Mitchell (2011)	232	MLQ-P	5	74.6	E	H	US
Hernández-Varas et al. (2019)	492	Ryff	5	1.6	E-M	H	ES
Hershberger and Walsh (1990)	49	PiL	20	59.2	E-O	H	US
Hicks et al. (2012)	221	Krause	8	100.0	E-O	H	US
Hill et al. (2019) Study 2	401	MILM-E	4	61.9	E-O	H	US
Hill et al. (2019) Study 2	401	MLQ-P	5	61.9	E-O	H	US
Hirsch et al. (2014)	66	SWBS-EWB	10	100.0	E-M	C	US
Hofer et al. (2014)	856	MLQ-P	5	58.6	M-V	H	I
Hofer et al. (2022)	101	Other	2	53.4	M-V	H	CH
Holahan (1995)	60	PiL	20	36.7	F	C	US
Hollis (1995)	161	SWBS-EWB	4	100.0	E-O	H	US
Holzer (2011)	58	SWBS-EWB	10	59.0	E-M	H	US
Homan (2016)	149	Ryff	6	72.5	F	H	US
Homan and Kong (2020)	3871	Ryff	7	55.4	F	H	US
Hong et al. (2018)	289	Ryff	7	61.2	E	H	US
Hooker and Masters (2014)	104	LET	6	71.1	E-O	H	US
Horwood and Anglim (2019)	539	Ryff	14	79.0	E-M	H	AU
Hughes (2018)	84	PiL	27	59.5	E-O	H	US
Hui and Fung (2009)	137	Ryff	9	65.0	E	H	HK
Hurd et al. (2014)	3334	Ryff+1	8	48.6	E^2^	H	US
Ishikawa (2013)	175	MLQ-P	5	58.0	F	H	US
Itzick et al. (2018)	501	MLQ-P	5	88.1	E-M	H	IL
Jaarsma et al. (2007)	294	PMP	39	72.1	F	C	NL
Jim, Richardson, et al. (2006)	167	MiLS	25	100.0	E-O	C	US
Johnny (2013)	158	SWBS-EWB	10	67.7	F	H	US
Johnson (2007)	644	LAPR-PMI	16	56.3	E-M	H	US
Junior (1999)	122	PiL	20	54.9	E-O	H	US
Kalafatoğlu (2022)	420	MLQ-P	5	68.3	E-M	H	TR
Kan (2007)	41	LAPR-PMI	16	32.0	M-O	C	US
Kang et al. (2021)	517	Ryff	7	44.9	E-O	H	US
Karataş et al. (2021)	1186	MLQ-F	10	57.8	E-O	H	TR
Kelley (2003)	91	LAP-PMI	16	77.0	E-O	H	US
Keresteš et al. (2012) Fathers	315	Ryff	9	0.0	E-M	H	HR
Keresteš et al. (2012) Mothers	350	Ryff	9	100.0	E-M	H	HR
Kim (2008) Care givers	157	PiL	20	81.5	E-O	H	KR
Kim (2008) Elders	157	PiL	20	45.2	M-V	H	KR
Kim et al. (2015) Females T1	233	FACIT-Sp-M	4	100.0	F	H	US
Kim et al. (2015) Males T1	136	FACIT-Sp-M	4	0.0	F	H	US
Kishida et al. (2004)	574	Ryff	14	74.6	E	H	JP
Klaas (1996)	77	PiL	20	78.0	O-V	H	US
Kleiman and Beaver (2013)	585	MLQ-P	5	82.0	E-M^2^	H	US
Klonover et al. (2023)	358	Krause	8	50.0	E-O	H	IL
Koenig et al. (2014)	129	PiL	20	70.0	F	C	US
Konkolÿ Thege et al. (2009)	364	PiL	20	53.6	F	H	HU
Kozar-Westman et al. (2013)	200	PiL	20		M-V	C	US
Krause (2009) Wave 4	1518	Krause	8	60.0	O-V	H	US
Krok et al. (2022)	333	MLQ-P	5	54.1	F	C	PL
Kulik et al. (2015)	611	LRI	28	50.9	E-O	H	IL
Kulik et al. (2017)	316	LRI	28	51.6	M-O	H	IL
Lawton et al. (2023)	141	SOMP-R	17	46.8	M-V	H	AU
Lebădă and David (2018)	28	PiL	20	75.0	O-V	C	RO
Lee (2017)	94	MLQ-P	5	67.0	E-M	H	HK
Lee et al. (2017)	228	Other	5	27.0	M-O	H	KR
Lee et al. (2022)	314	PiL	4	69.9	E	H	US
Lightsey and Sweeney (2008)	64	MLQ-P	5	88.0	E-M	H	US
Lin and Chan (2020) Study 1	301	MLQ-P	5	75.1	E	H	CN
Lin and Chan (2020) Study 2	527	MLQ-P	5	52.2	E	H	CN
Liu et al. (2020)	687	MLM	23	55.0	E-M	H	CN
Lopez (2020)	329	MLQ-P	5	38.0	E-O	H	US
Lu and Kao (2018)	301	LRI	6	54.2	O-V	H	TW
Lutzman and Sommerfeld (2021)	170	MLQ-P	5	0.0	M-V	H	IL
Lyke (2013)	168	MLQ-P	5	62.0	E	H	US
M. X. Zhang et al. (2018)	464	PiL	6	58.3	E	H	CN
Maki (2005)	79	LRI	28	100.0	M-V	H	US
Maqbool et al. (2021)	102	MLQ-P	5	60.8	E	H	IN
Markstrom et al. (1997) Study 1	244	PIES-P	8	100.0	E	H	CA
Markstrom et al. (1997) Study 2	153	PIES-P	8	58.8	E	H	CA
Martin et al. (2011)	154	PiL	20	50.0	E-M	C	US
Martos and Kopp (2012)	4841	BSCI-P	8	58.8	F	H	HU
Matheis et al. (2006)	75	SWBS-EWB	10	24.0	E-O	C	US
Mcgaffic (1995) Community	35	PiL	20	80.0	M-V	H	US
Mcgaffic (1995) Terminally ill	35	PiL	20	45.7	F	C	US
McManus et al. (2019)	184	MLQ-P	5	44.0	E-O	H	US
Mehnert and Koch (2008)	511	LAPR-C	8		F	C	DE
Meng et al. (2022)	765	MLQ-P	5		E-M	H	CN
Meraviglia (2006)	84	LAPR	48	100.0	M-O	C	US
Meyer (1998)	23	LAPR-PMI	16	39.1	E-M	C	US
Miller and Rottinghaus (2014)	229	MLQ-P	5	55.7	E	H	US
Miller-Lewis et al. (2019)	276	MLQ-P	5	95.6	F	H	AU
Mogollon (2021) Study 1	237	SFPS-S	16	56.5	E-O	H	US/CA
Mogollon (2021) Study 2	399	SFPS-S	6	51.4	E-O	H	US
Mogollon (2021) Study 2	399	MLQ-P	5	51.4	E-O	H	US
Mogollon (2021) Study 3	484	SFPS-S	6	44.4	E-O	H	US
Mohd Nazan (2017)	200	PiL	18	50.5	E-M	H	MY
Moisseron-Baudé et al. (2022)	100	MLQ-P	5	51.0	E-O	H	FR
Montag (2023)	955	MLQ-P	5	51.1	F	H	DE
Móró et al. (2011)	589	PiL	20	41.9	E-M^5^	H	HU
Morse (2021) Study 1a	945	Other	1	55.6	E-O	H	US
Morse (2021) Study 1b	846	Ryff	7	54.7	E-O	H	US
Morse (2021) Study 1b	846	Other	1	54.7	E-O	H	US
Morse (2021) Study 1b	846	Other	1	54.7	E-O	H	US
Morse (2021) Study 2	62	3DM-P	4	46.8	F	H	US
Morse (2021) Study 2	62	3DM-C	4	46.8	F	H	US
Morse (2021) Study 2	62	3DM-S	4	46.8	F	H	US
Mosalum (1998)	36	Ryff	3	61.0	E-M	H	BH
Moss et al. (2023)	259	MLQ-P	5	73.0	M-V	H	US
Mundaden and Tungol (2021)	123	MLQ-F	10	100.0	E	H	IN
Nakash et al. (2016)	110	MLQ-P	5	0.0	E	H	IL
Negri et al. (2020)	464	MLQ-P	5	54.7	E-M	H	IT
Neter et al. (2009)	92	PiL	18	72.8	E-O	C	IL
Newman (2022)	6732	MLQ-P	1	58.7	F	H	US
Newman et al. (2024)	1398	MLQ-P	3	60.3	E-O	H	US
Newton et al. (2024)	19619	MLQ-P	2	100.0	M-V	H	NZ
Ng and Yang (2023)	111	Ryff	10	64.5	M-V	H	SG
Nowicki et al. (2020)	325	MLQ-P	5	96.7	E-M	H	PL
O’Connor and Vallerand (1998)	128	Other	4	86.7	O-V	H	CA
Olesovsky (2003)	81	LRI	28	54.0	E-O	H	US
Owens et al. (2009)	174	MLQ-P	5	9.0	F	H	US
Park et al. (2010)	731	MLQ-P	5	71.0	E-O	H	US
Park et al. (2021)	542	MLQ-P	5	43.4	E-M	H	US
Peacock and Reker (1982)	63	LAP-P	9	55.6	E-M	H	GB
Pearson and Sheffield (1974) Females	80	PiL	20	100.0	E-O	C	GB
Pearson and Sheffield (1974) Males	64	PiL	20	0.0	E-M	C	GB
Pearson and Sheffield (1975) Females	97	PiL	20	100.0	E-O	C	GB
Pearson and Sheffield (1975) Males	84	PiL	20	0.0	E-O	C	GB
Pearson et al. (2015)	1277	LET	6	68.4	E	H	US
Pedaprolu et al. (2021)	622	Ryff	3	31.8	E-O	H	IN
Peebles (2006)	190	PiL	20	64.2	E-M	H	US
Peng (1994) Americans	339	Ryff	3		F	H	US
Peng (1994) Chinese Americans	132	Ryff	3		F	H	US
Peng et al. (2020)	355	MLQ-P	5	0.0	E	H	CN
Perryman (2003)	81	PiL	20	87.4	F	H	US
Petersen and Roy (1985)	318	Other	3		F	H	US
Pfund et al. (2022)	595	LET	6	56.0	O	H	US
Phillips and Ferguson (2013)	185	MLQ-P	5	56.8	O-V	H	AU
Pinquart and Frohlich (2009)	163	PiL	14	43.0	F	C	DE
Plach et al. (2003)	156	Ryff	14	100.0	M-V	C	US
Pollet and Schnell (2017)	475	SoMe-M	5	43.0	F	H	AT
Pulopulos and Kozusznik (2018) Study 1	172	LET	6	52.3	E-M	H	US
Pulopulos and Kozusznik (2018) Study 2	257	Ryff	9	51.7	E-M	H	US
Ramos (2012)	691	Ryff	9	46.7	E-O	H	US
Rasheed et al. (2022)	302	MLQ-P	5	63.9	E	H	PK
Ratner and Burrow (2017)	252	Ryff	9	57.4	E-O	H	US
Ratner et al. (2022)	1454	LET	6	46.0	E-O	H	US
Reker (1977)	48	PiL	20	0.0	E-M	H	CA
Reker and Cousins (1979)	248	PiL	20	76.2	E	H	CA
Reker and Peacock (1981)	63	LAP-P	9	0.0	E	H	US
Reker and Woo (2011)	120	LAP-P	8	51.2	M-V	H	CA
Reker and Woo (2011)	120	LAP-C	8	51.2	M-V	H	CA
Reynolds et al. (2022)	797	MLQ-P	5	77.8	E	H	US
Rice-Erso (1988)	99	PiL	10	100.0	E-O	C	US
Riedford (1997)	118	PiL	10	67.8	E-M	H	US
Robinson (2022)	71	LET	6	14.1	F	H	US
Robiou-Krischer (2008)	100	SWBS-EWB	10	100.0	E-O	H	US
Roche (2021)	77	Ryff	14	56.3	O-V	H	US
Russo-Netzer et al. (2019) Israelis	179	MLQ-P	5	52.0	E-M	H	IL
Russo-Netzer et al. (2019) Non-Israelis	86	MLQ-P	5	75.6	E-M	H	IL
Salt et al. (2017)	199	Ryff	14	100.0	M-O	H	US
Schettino (2012)	890	Ryff	7	56.5	F	H	US
Schlegel et al. (2011) Study 2	304	MLQ-P	5	86.2	E-O	H	US
Schnell (2009)	603	SoMe-M	5	53.0	F^4^	H	AT
Schnell and Danbolt (2023)	974	SoMe-M	5	51.0	F	H	DE
Schnell and Krampe (2020)	1522	SoMe-M	5	65.0	F	H	DE / AT
Schuurmans-Stekhoven (2018)	398	Ryff	7	49.0	M-V	H	JP
Scott (2007)	52	PiL	20	58.0	E-O	C	US
Shao et al. (2014)	214	LAP-P	3	40.2	M-V	C	CN
Shelton (2018)	330	MLQ-P	5	71.2	M-O	H	US
Shelton (2018)	330	PiL	7	71.2	M-O	H	US
Shenkman et al. (2023)	121	Ryff	8	0.0	M-V	H	IL
Sherman et al. (2011)	147	LRI	28	72.8	M-V	H	US
Shiri et al. (2020) Hospice	35	MLQ-F	10	87.0	E-O	H	IL
Shiri et al. (2020) Rehabilitation	36	MLQ-F	10	83.0	E-M	H	IL
Shrira et al. (2011)	608	MLQ-P	5	56.0	E-O^4^	H	IL
Siegel (2015)	54	FACIT-Sp-M	4	43.0	F	C	US
Sinclair et al. (2016)	393	MLQ-P	5	27.3	E-M	H	US
Singer et al. (2019)	292	Ryff	10	86.0	E-M	H	US
Singh et al. (2013)	210	Ryff	9	0.0	E-M	H	IN
Sipowicz et al. (2023)	46	LAPR-PMI	16	52.2	E-M	H	PL
Sipowicz et al. (2023) Asperger syndrome	41	LAPR-PMI	16	7.3	E-M	C	PL
Sipowicz et al. (2023) Asperger syndrome & depression	43	LAPR-PMI	16	27.9	E-M	C	PL
Sipowicz et al. (2023) Depression	40	LAPR-PMI	16	62.5	E-M	C	PL
Skarra (2014)	66	MiLS	21	62.1	M-V	C	US
Smith et al. (2010) Study 2	259	Ryff	7	64.0	E	H	US
Snow Lono (1993)	45	LAPR-P	8	66.7	F	H	CA
Soleimani et al. (2018)	300	SWBS-EWB	10	57.3	F	C	IR
Song et al. (2016)	305	FACIT-Sp-M	4	56.9	E-M	C	KR
Sosik and Dworakivsky (1998)	64	PiL	5	15.0	E-M	H	US
Sougleris and Ranzijn (2011)	227	Ryff	14	48.0	M-V	H	AU
Spitzenstätter and Schnell (2022)	98	SoMe-M	5	70.0	E-M	H	AT
Stanfill (2018)	344	SWBS-EWB	10	62.4	E-O	H	US
Staton et al. (2003)	661	SWBS-EWB	9	0.0	E-M	H	US
Stauner (2013) Early quarter	290	MLQ-P	5	69.0	E-M^2^	H	US
Stauner (2013) End of quarter	270	Ryff	9	69.0	E-M^2^	H	US
Steger et al. (2006) Study 1b	154	MLQ-P	5	70.0	E-M	H	US
Steger et al. (2006) Study 3	70	MLQ-P	5	63.0	E-M	H	US
Stockman (2008)	116	PiL	20	58.0	M	H	US
Stockman (2008)	116	LRI	28	58.0	M	H	US
Stolovy et al. (2009)	60	PiL	20	18.3	E-M	C	IL
Stroope et al. (2013)	1648	Other	1	53.0	F	H	US
Sumner (2017)	511	LET	6	58.9	E-O	H	US
Sun et al. (2022)	852	MLQ-P	4	42.4	E	H	CN
Sutin et al. (2022) - ELSA	8844	Other	1	56.1	F	H	GB
Suzman-Schwartz (2001)	104	LRI	28	30.0	E-M	C	US
T. Wang et al. (2023) Sample 6	478	CPS	12	80.8	E	H	CN
T. Wang et al. (2023) Sample 6	478	PiL	4	80.8	E	H	CN
T. Wang et al. (2023) Sample 6	478	Ryff	14	80.8	E	H	CN
Taubman-Ben-Ari and Weintroub (2008) Nurses	66	PiL	20	98.5	E-M	H	IL
Taubman-Ben-Ari and Weintroub (2008) Physicians	58	PiL	20	50.0	E-M	H	IL
Teas et al. (2022)	235	Ryff	7	80.0	M-V	H	US
Thill et al. (2020)	165	MLQ-P	5	54.6	E-M	H	LU
Thomas (2023)	210	MLQ-P	5	0.0	E-M	H	US
Thomas (2023)	210	MEMS-C	5	0.0	E-M	H	US
Thomas (2023)	210	MEMS-P	5	0.0	E-M	H	US
Thomas (2023)	210	MEMS-M	5	0.0	E-M	H	US
Tigershtrom and Boyraz (2022)	227	EMAS-R	12	79.3	E-O	H	US
Tigershtrom and Boyraz (2022)	227	MLQ-P	5	79.3	E-O	H	US
Triadó et al. (2007)	422	Ryff	9	52.6	O-V	H	ES
Trott (1996)	184	SWBS-EWB	10	37.0	E-M	H	US
Trzebiński et al. (2020)	306	Other	8	74.8	E-M	H	PL
Tsang (2021)	252	MLQ-P	5	52.4	F	H	HK
Türkarslan et al. (2020) Females	156	PiL	NR	100.0	E-M	H	TR
Türkarslan et al. (2020) Males	73	PiL	NR	0.0	E-M	H	TR
Updegrove (2019)	76	PiL	4	97.4	E-M	H	US
Usta and Bayram (2021)	72	MLQ-P	5	27.8	M-V	H	TR
Van Der Wende (2000)	50	Ryff	20	84.0	E-O	C	US
van Doornik et al. (2021) Control	63	MEMS	15	100.0	E^4^	H	NL
van Doornik et al. (2021) Experimental	65	MEMS	15	100.0	E^4^	H	NL
van Doornik et al. (2023)	112	MLQ-P	5	100.0	E	H	NL
van Doornik et al. (2023)	112	MEMS	15	100.0	E	H	NL
Van Orden et al. (2012) T1	65	GSIS-M	8	60.0	M-V	C	US
Van Tilburg (2021)	1316	ELQ-M	7	52.0	M-V	H	NL
Vanhooren et al. (2016)	337	MLQ-P	5	14.5	E-O	H	BE
Vanhooren et al. (2022)	358	MILM-E	4	82.4	F	H	BE
Vardeman (2017)	116	LET	6	82.0	E-M	H	US
Vehling et al. (2012)	178	LAPR-PMI	16	46.7	F	C	DE
Velasco-Gonzalez and Rioux (2014)	133	SWBS-EWB	7	63.2	M-V	H	FR
Vermote et al. (2023)	693	MLQ-P	3	62.1	O-V	H	BE
Wang et al. (2022)	245	MLQ-P	5	72.7	E-O	H	CA
Warner and Williams (1987)	234	ML	15	59.8	M-V	C	CA
Warner and Williams (1987)	188	PiL	20	59.8	M-V	C	CA
Wettstein et al. (2015)	89	Ryff	9		V	H	DE
Wheaton (1972) Inmates	78	PiL	20	0.0	E-M^2^	H	US
Wineman (1990)	118	PiL	20	67.8	E-O	C	US
Wingo et al. (2020)	5441	Ryff	10	70.6	F	H	US
Wnuk et al. (2012)	50	PiL	20	92.0	F	C	PL
Woo (2011) Study 1	228	LAPR-PMI	6	61.0	M-V	H	US
Wu et al. (2013)	165	PiL	6	55.0	E	H	CN
Yakushko (2004)	691	Ryff	9	100.0	F	H	US
Yampolsky et al. (2008)	85	SWBS-EWB	10		F	C	US/CA
Yeung and Breheny (2019)	452	LET	6	45.5	M-V	C	NZ
Yeung et al. (2019)	352	LET	6	14.0	M-V	H	NZ
Yeung et al. (2022)	380	LET	6	50.3	M-V	C	NZ
Yiu-kee and Tang (1990)	238	PiL	20	52.1	E-M	H	HK
Yoon et al. (2015)	450	MLQ-P	5	75.3	E-O	H	US
Yoon et al. (2021)	132	MLQ-P	5	71.2	E-M	H	US
Yu et al. (2016) Asian American	107	MLQ-P	5	57.0	E	H	US
Yu et al. (2016) European American	131	MLQ-P	5	69.5	E	H	US
Yu et al. (2017) Females	132	MLQ-P	5	100.0	E	H	US
Yu et al. (2017) Males	117	MLQ-P	5	0.0	E	H	US
Zambianchi and Carelli (2018)	245	Ryff	7	58.0	M-V	H	IT
Zeitchik (2000)	107	PiL	20	62.0	E-O	H	US
Zhang and Zhang (2017)	720	Krause	8	63.3	M-V	H	CN
Zhang et al. (2019) Wave 1	283	PiL	6	60.4	E	H	CN
Zhao et al. (2022)	705	MLQ-P	5	31.6	E-M	H	CN
Zhao and Zhang (2022)	1213	MLQ-F	10	72.7	E	H	CN
Zheng and Wang (2020)	309	MLQ-P	5	70.2	E-O^6^	H	CN
Zheng and Wang (2022) Study 2	346	MLM-Pr	5	49.4	E^1^	H	CN
Zheng and Wang (2022) Study 2	346	MLM-V	4	49.4	E^1^	H	CN
Zheng and Wang (2022) Study 2	346	MLM-Pu	4	49.4	E^1^	H	CN
Zhou et al. (2021)	204	Krause	4	44.1	M-O	H	CN
Zhou et al. (2022)	538	MLQ-P	5	53.2	E-O	H	CN
Zhu et al. (2022)	291	MMILS-MIL	4	59.1	E	H	CN
Zuckerman (2017)	172	MLQ-P	5	74.4	F	H	US

Notes.

1. Minimum age = 15.

2. Minimum age = 17.

3: Minimum age = 12.

4. Minimum age = 16.

5. Minimum age = 13.

6. Minimum age = 14.

**Table 3:** Heterogeneity of Between-Study Variance (Level 3)

Full	df	AIC	BIC	AICc	logLik	LRT	*p*	*QE*
	3	−494.20	−482.23	−494.14	250.01			3092.15
Reduced	2	−457.28	−449.30	−457.25	230.64	38.92	<.0001	3092.15

**Table 4:** Regression Model Results for Type of Purpose Scale as Moderator

Moderator	Studies	Beta	*SE*	*t*	*p*	95% CI	
						*LL*	*UL*
Other (Intercept)	99	.02	.01	1.67	.095	−.00	.05
EWB	21	.04	.03	1.17	.243	−.03	.10
Krause	6	−.01	.05	−0.28	.780	−.11	.09
LAP	18	.03	.04	0.75	.451	−.05	.11
LET	16	.04	.03	1.19	.233	−.02	.10
LRI	11	.03	.04	0.68	.505	−.05	.10
MLQ-P	92	.06	.02	3.56	**< .001**	.03	.09
PiL	77	.04	.02	1.96	.050	−.00	.08
Ryff	56	−.02	.02	−1.14	.254	−.06	.02
SoMe	5	.17	.06	2.99	**.003**	.06	.27

**Table 5:** Mean Effects for Type of Purpose Scale as Moderator

Moderator	Studies	Mean Effect (*Z_r_*)	*SE*	*t*	*p*	95% CI	
						*LL*	*UL*
Other	99	.02	.01	1.67	.095	−.00	.05
EWB	21	.06	.03	2.02	.044	.00	.11
Krause	6	.01	.05	0.14	.887	−.09	.10
LAP	18	.05	.04	1.32	.187	−.03	.13
LET	16	.06	.03	2.04	.042	.00	.11
LRI	11	.05	.04	1.29	.197	−.02	.12
MLQ-P	92	.08	.01	6.33	< .001	.06	.11
PiL	77	.06	.02	3.65	< .001	.03	.09
Ryff	56	.00	.02	0.05	.963	−.03	.03
SoMe	5	.19	.05	3.47	< .001	.08	.29

**Table 6:** Regression Model Results for Age Group as Moderator

Moderator	Studies	Beta	*SE*	*t*	*p*	95% CI	
						*LL*	*UL*
Emerging to Very Old (Intercept)	72	.07	.02	4.46	**< .001**	.04	.09
Emerging	60	−.03	.02	−1.51	.132	−.08	.01
Emerging to Middle	87	.02	.02	1.15	.252	−.02	.06
Emerging to Old	89	.03	.02	1.52	.129	−.01	.07
Middle	5	−.05	.07	−0.70	.487	−.18	.09
Middle to Old	12	−.04	.04	−0.99	.324	−.12	.04
Middle to very Old	53	−.11	.02	−4.59	**< .001**	−.15	−.06
Old to Very Old	23	−.16	.03	−5.00	**< .001**	−.22	−.09

**Table 7:** Mean Effects for Age Group as Moderator

Moderator	Studies	Mean Effect (*Z_r_*)	*SE*	*t*	*p*	95% CI	
						*LL*	*UL*
Emerging to Very Old	72	.07	.02	4.46	**< .001**	.04	.09
Emerging	60	.03	.02	1.92	.055	−.00	.06
Emerging to Middle	87	.09	.01	6.20	**< .001**	.06	.12
Emerging to Old	89	.10	.01	6.86	**< .001**	.07	.12
Middle	5	.02	.07	0.27	.790	−.11	.15
Middle to Old	12	.03	.04	0.66	.510	−.05	.10
Middle to very Old	53	−.04	.02	−2.31	**.021**	−.08	−.01
Old to Very Old	23	−.09	.03	−3.29	**.001**	−.15	−.04

**Table 8:** Regression Model Results for Region as Moderator

Moderator	Studies	Beta	*SE*	*t*	*p*	95% CI	
						*LL*	*UL*
Americas (Intercept)	224	.06	.01	6.28	**< .001**	.04	.08
Africa	7	−.09	.05	−1.74	.084	−.19	.01
Europe, UK & Ireland	79	−.00	.02	−0.02	.983	−.04	.04
Australia & New Zealand	12	−.04	.04	−1.16	.246	−.12	.03
Asia & Middle East	75	−.06	.02	−3.08	**.002**	−.10	−.02
Global	4	.06	.08	0.73	.464	−.10	.22

**Table 9:** Mean Effects for Region as Moderator

Moderator	Studies	Mean Effect (*Z_r_*)	*SE*	*t*	*p*	95% CI	
						*LL*	*UL*
Americas	224	.06	.01	6.28	**< .001**	.04	.08
Africa	7	−.03	.05	−0.55	.580	−.13	.07
Europe, UK & Ireland	79	.06	.02	3.87	**< .001**	.03	.09
Australia & New Zealand	12	.02	.04	0.51	.611	−.05	.09
Asia & Middle East	75	.00	.02	0.25	.804	−.03	.04
Global	4	.12	.08	1.49	.137	−.04	.29

**Table 10:** Multiple Moderator Model

Moderator	Beta	*SE*	*t*	*p*	95% CI	
					*LL*	*UL*
Intercept	0.02	0.02	1.12	.263	−0.02	0.06
EWB	−0.00	0.03	−0.05	.959	−0.06	0.06
Krause	0.07	0.05	1.45	.147	−0.02	0.16
LAP	0.01	0.04	0.34	.737	−0.06	0.09
LET	0.03	0.03	0.90	.369	−0.03	0.09
LRI	0.04	0.04	0.98	.328	−0.04	0.12
MLQ-P	0.04	0.02	2.63	**.009**	0.01	0.07
PiL	0.03	0.02	1.67	.095	−0.01	0.07
Ryff	−0.04	0.02	−2.44	**.015**	−0.07	−0.01
SoMe	0.12	0.06	2.12	**.035**	0.01	0.23
Emerging	−0.01	0.02	−0.23	.820	−0.05	0.04
Emerging to Middle	0.05	0.02	2.29	**.023**	0.01	0.08
Emerging to Old	0.05	0.02	2.29	**.023**	0.01	0.08
Middle	−0.04	0.07	−0.61	.543	−0.17	0.09
Middle to Old	−0.01	0.04	−0.33	.738	−0.09	0.06
Middle to very Old	−0.09	0.02	−3.58	**< .001**	−0.13	−0.04
Old to Very Old	−0.14	0.03	−4.46	**< .001**	−0.20	−0.08
Africa	−0.06	0.05	−1.30	.196	−0.15	0.03
Europe, UK & Ireland	0.03	0.02	1.48	.139	−0.01	0.08
Australia & New Zealand	0.02	0.04	0.70	.487	−0.04	0.09
Asia & Middle East	0.01	0.03	0.16	.871	−0.06	0.07
MTAS	0.00	0.00	2.27	**.024**	0.00	0.00
Indulgence	0.00	0.00	1.77	.077	0.00	0.00
